# Application of Piszkiewicz model on the electron transfer reaction of dithionite ion and bis-(2-pyridinealdoximato)dioxomolybdate(IV) complex

**DOI:** 10.1038/s41598-022-24096-7

**Published:** 2022-12-22

**Authors:** I. U. Nkole, S. O. Idris, I. Abdulkadir, A. D. Onu

**Affiliations:** 1 Department of Chemistry, Faculty of Physical Sciences, Ahmadu Bello University, Zaria, Nigeria; 2 Department of Chemistry, Faculty of Sciences, Federal College of Education, Zaria, Nigeria

**Keywords:** Chemical biology, Chemistry

## Abstract

The need to better understand the binding mode of antioxidants (sulfur oxyanions) kinetically is a concern in medicine. Hence, a spectrophotometric method was used to study the application of the Piszkiewicz model on the electron transfer reaction of dithionite ion (S_2_O_4_^2−^) and bis-(2-pyridinealdoximato)dioxomolybdate(IV) complex at 303 K and an absorption maxima of 560 nm. It follows an acid dependent reductive pathway that is medium sensitive. Charge distribution from the reaction species contributes to the redox efficiency of the system, resulting in a primary salt effect (NaCl) with an enhanced reaction rate. Alteration of the reaction medium with ethanol led to an elevation of reduction time as the charge catalysis was distorted by a drop in the system permittivity. Likewise, sodium dodecyl sulfate in the system decreased the reduction rate of the complex due to the low impact of hydrophobic and ion interaction between the micelle and substrates. First order reaction kinetics was observed in the concentration of the redox partners and a 2:1 (complex: S_2_O_4_^2−^) stoichiometry was obtained with the involvement of hydrogenated sulfite radical which resulted in the formation of sulfur dioxide and a Mo^2+^ deactivated complex. The occurrence of counterion catalysis is pronounced in the reaction system owing to the participation of like-charged substrates in the rate-controlling phase. The standard enthalpy (69.12 $\pm$ 0.05 kJ mol^−1^) and Gibbs energy (80.10 $\pm$ 0.07) kJ mol^−1^ suggest that the process is endothermic dependent. The investigation of the anionic surfactant effect on the reaction medium was quantitatively ascertained from the Piszkiewicz model of the complex interaction sequence.

## Introduction

Oxyanions with sulfur have a rich redox activity in cell metabolism and regeneration. Research have shown that their reactivity is medium sensitive as their antioxidant property is concerned^1^. The dithionite ion is a good reducing agent with an S–S linkage that has application in the pulp and paper industry. The rich chemistry of dithionite ion could be connected to its sulfur to sulfur linkage that is capable of promoting electron cloud within its host or system^2^. In the view of the above, some studies have been done to kinetically probe its reactivity. Pal and Gupta^3^ observed that hydrogen ions had a limiting impact on the rate of the reduction of hexachloroplatinate(IV) with dithionite over the range of pH studied. The change in solution concentration and relative permittivity increased the rate with a negative entropy, and the participation of free radical was positive. Ukoha et al*.*^4^ noted nonexistence of catalysis with added cation and anion, an increment in redox rate with a rise in [H^+^], and an invariant in reaction rate of reduction of adipato bridge iron(III)-salen complex with S_2_O_4_^2−^ as ion concentration and system medium permittivity varied. Gupta et al.^5^ recorded free radical generation and intermediate complex formation, and S–S bond cleavage to give 2HSO_3_^−^ ion for the reduction of tris-(pyridine-2-carboxylato)manganese(III) by dithionite in sodium picolinate-picolinic acid buffer medium.

The chemistry of complexation of molybdenum from higher to lower oxidation states is of great interest due to its role as enzymes in nitrogen metabolism and as a vital micronutrient in lower animals. It can be found in soybeans, chicken liver, cow milk, rabbit liver, hog liver, bovine liver, azotobacter, and coli in different forms of enzymes^6^. We aim to probe the likely roles of molybdenum in enzymes by building a model system that will give insight into some of the properties of molybdenum in enzymes and generate kinetic data that will explain its redox property. Also, the understanding of its redox reaction in a regulated ion-medium will be a plus to its chemistry.

An anionic surfactant (sodium dodecyl sulfate, *SDS*) has applications in many cleaning and hygiene products and is frequently used as a constituent for solubilizing cells throughout RNA and/or DNA isolation, as well as for denaturing proteins^7^. Its application in chemical industries is centred on the catalysis of organic and inorganic molecular reactions due to ionic interaction between substrates or intermediates (cationic species) and the surface of the micelle, resulting in layer contact’s increase among the containing reactant molecules. Its aggregates inhibited the oxidation of *DL*-aspartic acid slightly^8^, acetophenone^9^, dextrose^10^, and cyclopentanone^11^. The Piszkiewicz model assumes a catalytic micelle generated by surfactant monomers and its treatment in redox reactions is used to check the dependency of the rate constant on the surfactant concentration and is useful in examining reaction mechanistic routes^12^. The enhancement of the reaction rate depends on increased local concentration of the reactants at the surface and in the interior of the micelle, stabilisation of the transition state of the reaction, and medium effects (polarity, microviscosity, and charge effects inside the micelle)^13^. The applicability of the Piszkiewicz model to divers substrate–micelle systems has been reported. The oxidation of thioglycolic acid with salicylidene-manganese(III) complex in the presence of *SDS* showed that the existence of pre-micellar composites and hydrophobic interaction contributed positively to the redox process^14^. The fading of nitrophenol violet with hydroxide ions in the presence of cationic and anionic surfactants revealed nitrophenol violet’s responsiveness for investigating nucleophilic attack systems sponsored by electrostatic phenomenon^15,16^. Also, the oxidation of methyl violet with hydroxide ions in the presence of cationic and anionic surfactants showed that high binding affinity which is provided by electrostatic and hydrophobic interactions assisted the degradation of the dye^12^.

The resultant SDS’s influence on the reaction kinetics is quantitatively determined with the Piszkiewicz kinetic model that shows the extent of interaction between the micelle and the substrates^17^. This model will further reveal the cooperativity status and binding affinity of molybedenum and sulfur-containing reductant with the aid of sodium dodecyl sulfate.

## Experimental

The synthesis and characterization of the bis-(2-pyridinealdoximato)dioxomolybdate(IV) ion ([Mo^IV^O_2_(paoH)_2_]^2−^) was achieved by engaging the synthetic approach of Konidaris et al.^18^ and fourier transform infrared spectrophotometer (FTIR-8400S Shimadzu, Double Beam) and ultraviolet–visible (Cary 300 Series UV–Vis Spectrophotometer, Agilent Technologies, USA) correspondingly. The complex ion was synthesized using 2-pyridinealdoxime and molybdenum(IV) oxide gotten from Sigma Aldrich, Germany. Dithionite ion and hydrochloric acid obtained from Merck were used as a reductant and proton (H^+^) generator respectively. Change in the influence of salt was investigated by the use of NaCl obtained from the British Drug House, Nigeria. Variation in the reaction medium permittivity was carried out with ethanol (BDH, Nigeria). Sodium dodecyl sulfate (Sigma Aldrich, Germany) was used to generate a micelle in the reaction system. The counterion catalysis was studied by using ammonium chloride and sodium formate gotten from British Drug House, Nigeria. Methanol with acrylamide (Merck) was used to check the involvement of unstable molecules with unpaired electron in the redox system. Filter paper, K_2_Cr_2_O_7_ (Merck), H_2_SO_4_ (BDH), SnCl_2_ (BDH), and KSCN (Merck) were used in the product examination.

The spectrophotometric approach of mole ratio analysis was applied to the stoichiometry of the reaction as archived earlier^19–21^ at stable temperature (303 K), salt effect, and 560 nm. The breaking position on the curve of Abs versus mole ratio was gotten from the absorbance recorded within 600 min. The product mixture that gave the breaking point was used to classically examine the compounds/ions formed. The appearance of molybdenum(II) ion at the end of the oxidation–reduction process was determined by adding 0.1 cm^3^ of concentrated H_2_SO_4_, heating to a thick white haze, and cooling. Following that, 0.5 cm^3^ distilled H_2_O, 0.5 cm^3^ KSCN (1.0 mol dm^−3^), and one drop of acidified 0.25 mol dm^−3^ SnCl_2_ solutions were added^22,23^. The presence of SO_2_ was also tested by dipping a filter paper into an acidified 0.5 mol dm^−3^ K_2_Cr_2_O_7_ solution and then transferring it into the solution of the product mixture^23^.

The order with respect to the concentrations of the redox partners was determined from rate data obtained from the variation of absorbance with time as the concentration of the reaction mixture decreased using an ultraviolet visible spectrophotometer (Model 721 PEC Medical) at a 560 nm absorption maxim^24–26^. The investigation of change in salt effect (µ), concentration of acid, and reaction medium permittivity (D) was done by varying them while retaining the concentration of other parameters^27–29^. The slope of the graph of lnA against time was used to calculate the 1st order kinetic constant, and the arithmetic ratio of *k*_*obd*_ with [S_2_O_4_^2−^] was used to calculate the 2nd order rate constant (*k*_2_). The contributions of unstable molecules in the redox process were studied by introducing 0.4 cm^3^ acrylamide to the reacting mixture with surplus methanol^30,31^. Thermodynamic parameters were investigated by varying the temperature of the reaction mixture with Grant JB1 thermostated H_2_O immersion, and the data generated was evaluated with Eyring approach to evaluating activated parameters^32–34^. The surfactant effect on the reaction rate was explored quantitatively by using the Piszkiewicz equation (Eq. 1).

1 $$k_{{obd}} = \frac{{k_{M} \left[ D \right]^{n} + ~k_{W} K_{D} }}{{\left[ D \right]^{{n~}} + ~K_{D} }}$$

where k_m_ and k_w_ represent the kinetic constants in the presence and absence of surfactant correspondingly. n represent the amount of micelle (D) and K_D_ denote the detachment constant of the surfactant molecules to their unrestricted state. [*D*] denote the [surfactant] while [*D*]^n^ is obtained by subtracting critical micelle concentration (CMC) from [*D*]. *k*_*obd*_ designate the 1^st^ kinetic constant^12,15^.

## Results and discussion

**Figure 1:**
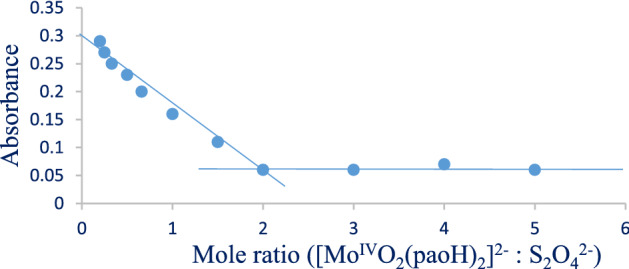
Reaction stoichiometry of [Mo^IV^O_2_(paoH)_2_]^2−^ and S_2_O_4_^2−^ at [Mo^IV^O_2_(paoH)_2_^2−^] = 13.0 × 10^–4^ mol dm^−3^ and T = 303 K.

The relative mole ratio of the reacting redox species proceeds with a double-electron exchange from a single mole of the dithionite ion to a double mole of the molybdenum complex. It is supported by the mole ratio (2) breaking point in Fig. 1.

The consumption of one mole of dithionite ion by a double mole of the Mo(IV) complex resulted in the generation of sulfur dioxide and molybdenum(II) products. The change of the damped paper submerged in acidified K_2_Cr_2_O_7_ from orange to green points to the presence of SO_2_. The identification of molybdenum(II) ion at end of the redox activity is demonstrated by the formation of a red color after the inclusion of 0.5 cm^3^ KSCN (1.0 mol dm^−3^) and a single drop of acidified SnCl_2_ (0.25 mol dm^−3^), among other reagents (Eq. 2).2

**Figure 2:**
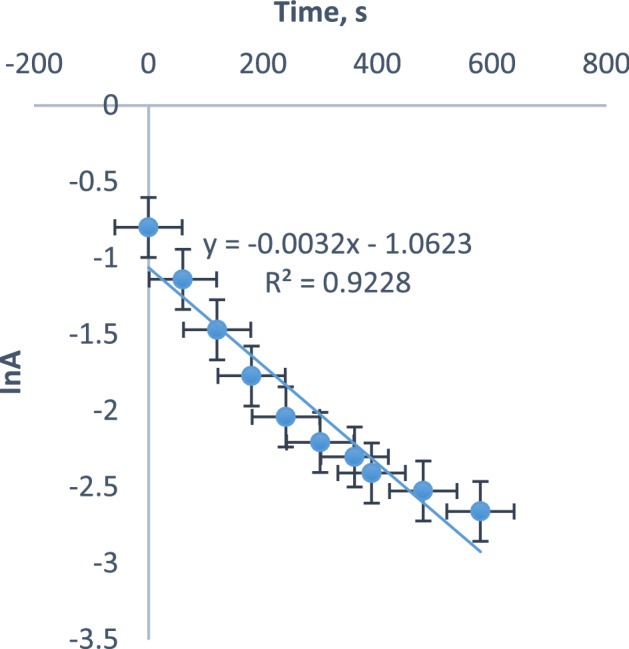
Nonlinear least square graph of InA against time for the reduction of [Mo^IV^O_2_(paoH)_2_]^2−^ by S_2_O_4_^2−^.

**Figure 3:**
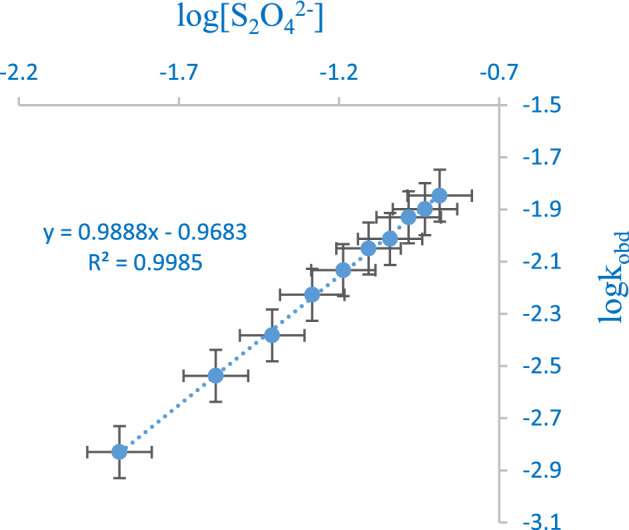
Logarithm plot of k_obd_ against [S_2_O_4_^2−^] for the reduction of [Mo^IV^O_2_(paoH)_2_]^2−^ by S_2_O_4_^2−^.

The order assumed by the complex and the dithionite ion concentrations are determined from the 1st order kinetics graph and the logarithm graph of the 1st kinetics constant with the concentration of the dithionite ion as shown in Figs. 2 and 3 respectively. The linearity of the pseudo-first graphs and the slope (0.9888) of the logarithm plot of *k*_*obd*_ with [S_2_O_4_^2−^] suggested a first order in [complex] and [S_2_O_4_^2−^] respectively. The consistency of *k*_*2*_ in Table 1 with respect to the change in the concentration of S_2_O_4_^2−^ ion reinforced the first order in [S_2_O_4_^2−^]. Also, the increase in acid concentration favored the protonation of S_2_O_4_^2−^ ion that portrayed a single acid dependent pathway. The observed acid catalysis eases the electron transfer by adequately polarizing the medium and providing an efficient link for interaction of the reactants. Thus, the oxygen exchange between dithionite ion and water is faster due to an enhanced reactivity of protonated dithionite ion^35^.

**Table 1:** Kinetics constants for the reduction of [Mo^IV^O_2_(paoH)_2_]^2−^ by S_2_O_4_^2−^.

10^3^[S_2_O_4_^2−^], mol dm^−3^	µ, mol dm^−3^	10^2^[H^+^], mol dm^−3^	10^3^*k*_*obd*_, s^−1^	10^2^*k*_*2*_, dm^3^ mol^−1^ s^−1^
13.00	0.50	1.60	1.48	11.38
26.00	0.50	1.60	2.90	11.16
39.00	0.50	1.60	4.15	10.63
52.00	0.50	1.60	5.94	11.42
65.00	0.50	1.60	7.37	11.33
78.00	0.50	1.60	8.93	11.44
91.00	0.50	1.60	9.72	10.68
104.00	0.50	1.60	11.75	11.29
117.00	0.50	1.60	12.63	10.79
130.00	0.50	1.60	14.23	10.95
65.00	0.50	1.60	7.37	11.33
65.00	0.50	2.00	7.83	12.05
65.00	0.50	2.40	8.98	13.81
65.00	0.50	2.80	10.36	15.94
65.00	0.50	3.20	11.05	17.06
65.00	0.50	3.60	12.21	18.77
65.00	0.50	4.00	13.59	20.90
65.00	0.50	4.40	14.74	22.68
65.00	0.50	4.80	15.43	23.74
65.00	0.50	5.20	16.25	25.16
65.00	0.30	1.60	2.76	4.25
65.00	0.35	1.60	3.45	5.31
65.00	0.40	1.60	5.29	8.14
65.00	0.45	1.60	5.98	9.21
65.00	0.50	1.60	7.37	11.33
65.00	0.55	1.60	8.75	13.46
65.00	0.60	1.60	10.59	16.29
65.00	0.65	1.60	12.66	19.48
65.00	0.70	1.60	14.50	22.32
65.00	0.75	1.60	16.35	25.15

The detected rise in reaction rate with change in salt effect on the reaction medium as shown in Table 1, suggests a positive salt effect that brings about the coming together of the negatively charged-like redox species^28^ (Eq. 6). The presence of dissolved charged ions in the reaction medium contributed to the electrolytic nature of the system that aided the acceleration of the reaction rate. This observation, is maintained by the reducing of reaction rate on the decrease in the medium polarity (permittivity) as shown in Table 2. The decrease in the medium permittivity decreases the charge to charge ratio interaction by making them less mobile in the system^24^.

**Table 2:** Effect of system permittivity on the reduction rate of [Mo^IV^O_2_(paoH)_2_]^2−^ by S_2_O_4_^2−^.

D	78.3	77.7	77.2	76.8	76.3	75.9	75.4	74.9	74.5	74.1
10^3^*k*_*obd*_ (s^−1^)	7.37	6.67	5.75	5.21	4.60	3.91	3.45	2.99	2.30	2.07
10^2^*k*_*2*_ (dm^3^ mol^−1^ s^−1^)	11.33	10.27	8.85	8.14	7.08	6.02	5.31	4.60	3.54	3.18

Counterion catalysis emanating from the introduction of ammonium ion into the reaction medium is notable for the reaction rate, suggesting the reactivity of like-charged species at the equilibrium collision state^14,30^ (Eq. 6). Meanwhile, the addition of formate ion inhibited the kinetic rate due to repulsive electrostatic interaction between the redox species (Table 3). The polymerization observed in the reaction system on the addition of monomeric acrylamide with excess methanol suggests the production of sulfite radical^35^ which was necessary in the reduction of the complex as displayed in Eq. (8).

**Table 3:** Effect of counterions on the reaction rate for the reduction of [Mo^IV^O_2_(paoH)_2_]^2−^ by S_2_O_4_^2−^.

Ion	10^2^[Ion], mol dm^3^	10^3^*k*_*obd*_, s^−1^	10^2^*k*_*2*_ dm^3^ mol^−1^ s^−1^	Ion	10^2^[Ion], mol dm^3^	10^3^*k*_*obd*_, s^−1^	10^2^*k*_*2*_ dm^3^ mol^−1^ s^−1^
NH_4_^+^	0.00	7.37	11.33	HCOO^−^	0.00	7.35	11.64
	1.50	8.52	13.10		1.50	6.22	09.42
	2.00	9.44	14.52		2.00	5.76	08.86
	2.50	10.13	15.39		2.50	5.06	07.76
	3.00	11.36	17.01		3.00	4.61	07.08
	3.50	12.43	19.13		3.50	3.92	06.02
	4.00	13.35	20.54		4.00	3.45	05.31
	4.50	14.04	21.61		4.50	2.53	03.89

**Figure 4:**
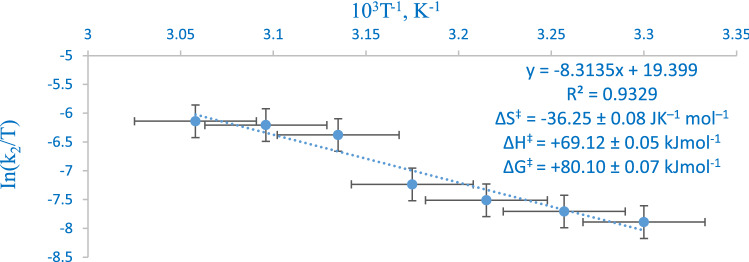
Rate dependent on temperature plot for the reduction of [Mo^IV^O_2_(paoH)_2_]^2−^ by S_2_O_4_^2−^.

The thermodynamic study of the reaction shows that the reaction rate was rapidly accelerated as temperature was elevated (Fig. 4) and it portrays a reaction where there is an associative route due to the mutual ordering of solvated molecules at the rate-determining step as shown by the value of ΔS^‡^ = − 36.25 $\pm \hspace{0.17em} 0.08$ JK^−1^ mol^–1^. The observed ΔH^‡^ and ΔG^‡^ (69.12 $\pm$ 005 and 80.10 $\pm \hspace{0.17em}0.$07 kJ mol^−1^) suggest a reaction scheme where there is a little or no energy loss during the associative processes that result in the formation of the energized activated complex^31^ (Eq. 6).

**Figure 5:**
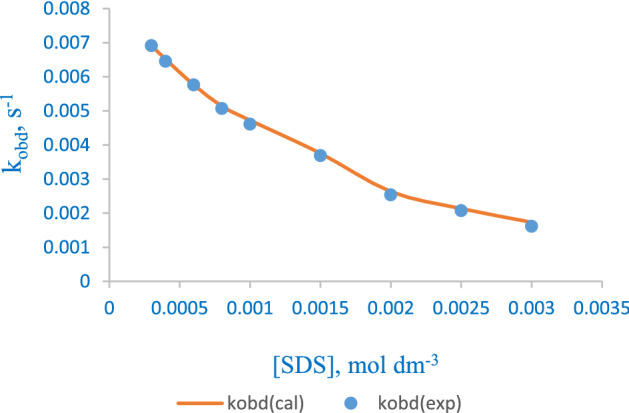
Plot of *k*_*obd*_ against [SDS] fitted into Piszkiewicz’s equation for the reduction of [Mo^IV^O_2_(paoH)_2_]^2−^ by S_2_O_4_^2−^.

**Scheme 1:**
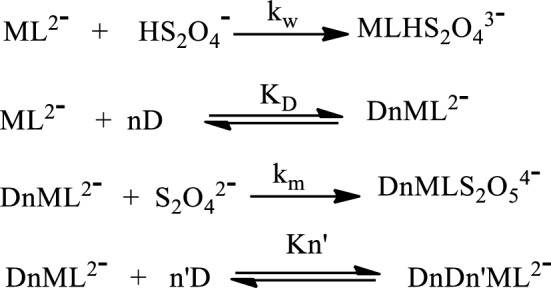
The micelle—substrate model of Piszkiewicz’s approach.

The SDS influence on the observed kinetic rate was negative as the redox partners are greatly influenced by repulsive electrostatic attraction in the presence of surfactant aggregates. This effect was evaluated by using the Piszkiewicz kinetic model that showed an effective binding (1/K_D_) and positive cooperativity (n > 1) between the substrate and the surfactant aggregates, leading to a deceleration in the reaction rate (Fig. 5). The binding and cooperativity of the surfactant with substrates was endothermically controlled and this could be attributed to the repulsive state of the negative charged SDS’s head with the negative charged substrates at the micelle's Palisade-Stern layer, which is thought to contain an intense molar [SDS] with little polarity. Hence, the probable location of the reaction is at the Gouy-Chapman layer (where counterions are unbound) with less repulsion effect^7,12,15,36^. The catalytic micelle—substrate model of Piszkiewicz’s approach for the reaction of molybdenum(IV) complex with dithionite ion is shown in Scheme 1. M^2−^ is the complex ion, Kn’ is the association constant of the additional interactions, and *n’* denote the additional number of SDS molecules. The parameters of Eq. (1) were obtained using a nonlinear least-squares concept as presented in Table 4.

**Table 4:** Parameters of Piszkiewicz model.

Surfactant	*N*	*K*_*D*_	1/*K*_*D*_	*k*_*m*_ (dm^3^ mol^−1^ s^−1^)	*r*^2^	*k*_*w*_ (dm^3^ mol^−1^ s^−1^)
SDS	1.442	0.2902	3.446	0.9613	0.9765	0.0128

The model (Scheme 1) depicts the mode of responsiveness of the surfactant with the substrates, wherein the complex comes into contact with the micelle to form a complex-micelle energized molecule in an equilibrium state. On the addition of the dithionite ion into the reaction system, the complex-micelle energized molecule interacts with it through the hydrophobic tail of the micelle, and hence, a redox process is executed among them. This process can proceed further by adding more surfactant and substrates to the reaction system.

**Scheme 2:**
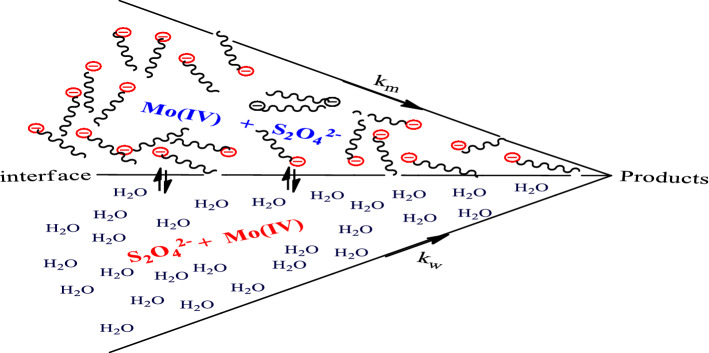
Piszkiewicz pseudo-phase reaction environment.

Scheme 2 describes the pseudo-phase reaction environment with an interface of micelle and water molecules. The interaction of Mo(IV) complex and S_2_O_4_^2−^ at the micelle environment is accompanied by k_m_, which is driven by the hydrophobic effect, and the aqua environment is associated with k_w_, which is ion-dependent (electrostatic effect)^7,12,15^.

Comparatively, the investigation of SDS micelle’s effect on the oxidation of L-lysine with AuCl_3_(OH)^−^ showed a pronounced inhibition on the oxidation rate. It was proposed that lysine molecules were linked to surfactant premicellar aggregates, and electrostatic repulsion occurred between SDS and Au(III), limiting redox species closeness in the Stern layer of the micelle^37^.The presence of SDS on the reaction of tris-(2,2′-bipyridyl)iron(II) and azidopentacyanocobaltate(III) complexes resulted in retardation of the reaction rate and the Piszkiewicz model reveals a good cooperativity pattern (n = 2.184) and poor binding of the reactant molecules on the micelles^36^. The influence of SDS on the oxidation of diphenyl sulfide with hydrogen peroxide enhanced the reaction rate, resulting in a sulfoxide product^38^.

**Scheme 3:**
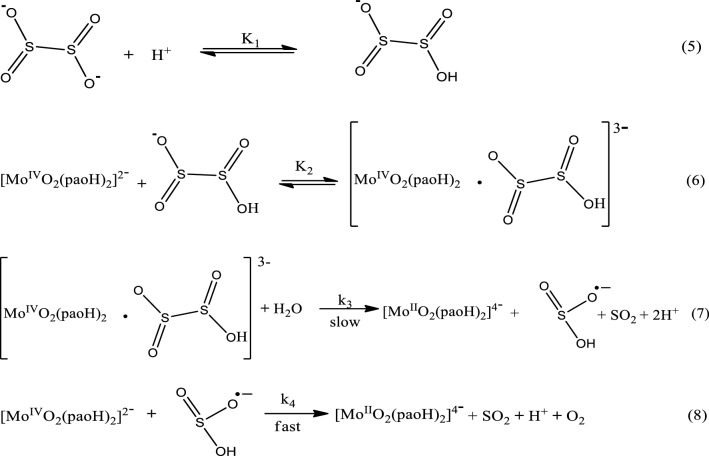
Elementary redox route of [Mo^IV^O_2_(paoH)_2_]^2−^ and S_2_O_4_^2−^.

On the basis of above data, the below reaction Scheme 3 is proposed for the reaction (Eqs. 5–8) which is analogue to the outer-sphere mechanistic route according to Taube mechanistic approach:

## Conclusions

The application of Piszkiewicz model on the electron transfer reaction of dithionite ion and bis-(2-pyridinealdoximato)dioxomolybdate(IV) complex was studied. The reaction featured a stoichiometry of 2:1 (complex: S_2_O_4_^2−^), signifying that the dithionite ion is a two-electron donor with a reactive sulfite radical specie. First order molecularity was observed in the concentration of the oxidizing and reducing agents. Acid and counterion catalysis prevailed on the reaction rate. The primary salt effect was in operation with an increment in the reaction rate and the medium permittivity buttressed the vitality of dissolved charged molecules in the reduction system for enhancing the kinetic rate. The activation parameters (ΔS^‡^, ΔH^‡^, and ΔG^‡^) reveal that the reaction is endothermically controlled with the establishment of an ordered activated complex. The anionic surfactant interacted with the substrates, leading to a drop in the kinetic rate. This effect could be attributed to the unbounding of counterions at the *Gouy-Chapman* region of the SDS aggregates. The binding constant (1.442) and cooperativity index (2.3293) obtained from Piszkiewick’s equation supported the resultant influence of the anionic surfactant on the kinetic rate. The observed reactivity between the negatively charged redox species in the presence of anionic surfactant reveals that the reaction is also electrostatically controlled. The observed electrostatic interactions have a vital significance in the assembling of the redox species despite the medium effect that may be in place. Repulsive electrostatic interaction prevailed over the hydrophobic interaction which led to reactivity in the reaction system and eventually stopping the reaction at a high concentration of the surfactant. The outcome of the study reveals the systematic bimolecular redox processes that take place in biological and chemical medium-sensitive systems. The Piszkiewicz model treatment fits the experimental data in showing that the micellar rate enhancement is due to the concentration of both redox species at the micelle-water interface.

## Data Availability

The datasets used and/or analysed during the current study available from the corresponding author on reasonable request.

## Abbreviations

SDS: Sodium dodecyl sulfate
RNA: Ribonucleic acid
DNA: Deoxyribonucleic acid
FTIR: Fourier Transform Infrared Spectrophotometer
BDH: British Drug House
D: Medium permittivity
µ: Salt concentration
k_obd_: Observed rate constant
k_2_: Second order rate constant
CMC: Critical micelle concentration
A/Abs: Absorbance
T: Temperature
K: Equilibrium constant
